# Provings and Clinical Observations with High Potencies

**Published:** 1894-01

**Authors:** Malcolm Macfarlan

**Affiliations:** Philadelphia, Pa.


### PROVINGS AND CLINICAL OBSERVATIONS WITH HIGH POTENCIES.

MALCOLM MACFARLAN, M. D., PHILADELPHIA, PA.

### TROMBIDIUM$^{5C}$.

Oppression of chest; thought her breath was leaving her.

### TRITICUM$^{5C}$.

Tightness and soreness in the middle of the sternum. It catches him every time he coughs; pain across the chest at the diaphragm.

Soreness down the sternum and in the epigastrium; pain going through to the back from the sternum; sneezing caused distress across the upper part of his chest.

### TRILLIUM$^{10M}$.

Aching pains through the chest; sensation as of cold in the chest.

### TEREBINTH.$^{17M}$.

Smothering feeling and pressure at ensiform cartilage; feels as if something were in the back part of wind-pipe smothering him whenever he lies down; tight cough after lying down.

### TRILLIUM-PEND.$^{10M}$.

Often seized with sudden pain at the end of the sternum, like cramp; severe sneezing; thought he would have died from suffocative attacks of irregular breathing, with sneezing.

### ZINC.$^{5C}$.

Dull, aching pain in chest; *coughs mostly at night;* raises much phlegm.

### HEART.

### CORNUS-FLORID.$^{45M}$.

Violent pain settled about the heart, causing a feeling of pressure and palpitation.

### CACTUS-GR.$^{CM}$.

Sensation as if the heart was compressed by a hand.

### CUPRUM-ACET.$^{45M}$.

Fearful griping and spasm about the region of the heart, lasting only a moment.

### IRIS$^{CM}$.

Distress commenced with beating, throbbing in and about the heart and centre of the sternum.

### KALI-HYD.$^{CM}$.

Fluttering of the heart when waking; fluttering of the heart on exertion; giddiness.

### SPIGELIA$^{75C}$.

Stitch or darting-like pain in the heart.

### STRONTIA$^{5C}$.

Sensation of smothering about her heart, with oppressed breathing; restless; heavy load at her heart; moaned continually; symptoms appeared on third day, taking medicine every hour.

These prominent symptoms verified several times.

### UPPER EXTREMITIES.

### ACONITE$^{50M}$.

Pains mostly in small joints of extremities.

### ADEPS$^{1M}$.

Her elbows painful.

### ÆSCULUS$^{56M}$.

Her hands swell up after washing in water.

### AGARIC$^{2M}$.

Arms stiff; muscles sore.

### AILANTHUS$^{45M}$.

A blister appeared on the end of the thumb, small sores around the finger nail.

### BURSA-PAST.$^{9M}$.

His fingers very painful; pain in his left shoulder so severe that he thought it was injured in some way; a felon appeared on the tenth day after he began taking the medicine.

### CAUST.$^{30M}$.

Fore arms in front very sore to touch and pressure; muscles of the extremities sore generally.

### CARB-SULPH.$^{45M}$.

Sudden, violent, spasmodic pain begins at the left elbow and extends down the whole left side to the foot.

### CHELON-GLAB.$^{45M}$.

Whole upper extremity was so sore she thought the parts were bruised and skin removed; elbow mostly affected.

### CICHOR-INTYB.$^{5C}$.

Rheumatic-like pain across the fingers; more in left hand and somewhat on fore-arm.

### CORNUS-FLORID.$^{45M}$.

Neuralgic sharp pains began in the right elbow extending to the hand and shoulder, passing down the right side and then up the left side; could not use the arm properly because of pain and lameness; hands and feet were swollen, and the pains were of a darting needle-like character.

Elbows and wrists much affected.

### COCHLEAR.$^{10M}$.

Grasping-like pain in both shoulders.

### CARB-VEG.$^{76M}$.

Severe pain from right shoulder to elbow; lameness.

### DRACONTIUM$^{10M}$.

Cured violent aching pain in her left shoulder. The symptom was chronic and correspondent to the proving.

### EUPAT-PERF.$^{CM}$.

Left ankle, left hip, and left shoulder pains more than other parts of body.

### GLANDERINE$^{12C}$.

Right arm pains in front and above the elbow; very sore to touch.

### GETTYSBURG$^{45M}$.

Aching mostly in both shoulders.

### HYPERICUM$^{45M}$.

In two provers caused severe pain in the last phlanges of the fingers, mostly thumb, fore, and little finger.

### MERC-VIV.$^{101M}$.

General aching; right shoulder mostly affected.

### MYRTUS$^{5C}$.

*Pains in the arm pits* and in the shoulder, as if the latter were out of place. The suffering was severe.

### PLANTAGO-MINOR$^{11C}$.

*Sharp pain in wrist joint;* both affected; little finger of the right hand seems stiff. Wrists and fingers more or less stiff.

*Sharp pain* suddenly appears at the middle of the left fore-arm; leaves a soreness.

Seized in the night with violent pain like rheumatism at the middle of the left arm; hands appear hot and are sensitive. These were symptoms common to many provers.

### PHYTOLAC.$^{47M}$.

Intense pain in and around right elbow joint, and down the fore-arm. She cries out because of it, constantly.

### RANUNCULUS-ACRIS$^{10M}$.

Burning sensation in the hands.

### RUMEX$^{45M}$.

Aching sensation from the shoulder-blades all the way down the back to the region of the kidneys. Muscles of the back affected.

### SILICA$^{CM}$.

Cures inflammation at the ends of the fingers, with offensive discharges; felon, onychia, paronychia. Often verified. Fistulous discharges from bone in like manner.

### TARAXACUM$^{67M}$.

In two cases caused aching in the left upper extremity, side of head and ear. Hot hands.

### LOWER EXTREMITIES.

### ACON.$^{50M}$.

Pain in finger joints.

### ÆSCULUS$^{56M}$.

Walking difficult. Weakness and aching in the legs.

### ADEPS$^{1M}$.

Great weakness in the posterior part of her knees. Rising from sitting position is painful. Her hips and elbows ache.

### AGARIC.$^{2M}$.

A good deal of pain over both hip joints. She gets up from a chair like an old woman. Joints seem stiff.

### APOCYNUM$^{6M}$.

General stiffness of the legs and joints of the body. Bending is painful. *Stiff knees* a prominent symptom in many provers.

### ARGENT.$^{45M}$.

Severe soreness in muscles, mostly of extremities.

### ARUM-TRI.$^{16M}$.

Her feet are painful on standing and pressure; even the stockings when drawn on pain her; feet feel bruised, and cause her great suffering.

### ARANS-EXCELS.$^{5C}$.

Aching through the hips on walking.

### AURUM-MUR.$^{5M}$.

Legs swollen, and very tender to touch, especially along the inner side of the tibia. Backache severe.

### BORAX$^{3M}$.

Cramps in the muscles of the calf of the leg; left mostly affected.

### BROMINE$^{CM}$.

Aching pain in both knee joints.

### BURSA$^{9M}$.

Toes are painful and sensitive.

### BROM.$^{CM}$.

Wonderfully curative in certain kinds of old varicose ulcers occurring in the aged and those broken down in general health.

### CAUST.$^{30M}$.

Sudden severe pain commences in the *left hip joint.* Lasts a short time. Feels as if it had been injured. Legs very sore, or rather the lower extremities ache and feel tired.

### COBALT$^{6M}$.

Tingling sensation in the feet.

### CARB-SULPH.$^{45M}$.

Sudden severe pain begins in the left elbow and arm, and extends down the whole left side to the foot.

### CIMICIFUGA$^{95M}$.

Acute pains through her lower limbs, something of the nature of growing pains, but a great deal worse; legs mostly affected; medicine given for three weeks twelve times daily.

### COCHLEARIA$^{40M}$.

Aching pain in or at the adductors of the thighs at their upper part. In the language of the prover, it almost drags her to pieces.

### CUPRUM-ACET.$^{45M}$.

Ankles and knees are painful; aching soreness.

### CUPRUM-ACET$^{45M}$.

Sensation of sickness down the inside of the left thigh and leg from the hip to the foot; rest of the extremities less so. Right lower extremity less affected.

### ERECTHITES$^{10M}$.

Lower limbs seem stiff and painful.

### EUPAT-PERF.$^{CM}$.

Sharp pain in the right heel as if it had been injured.

Sharp burning pain in the feet; had to remove her shoes while the pain lasted; feet seemed swollen.

Left hip and ankle are troubled with sharp pains which come on instantly and go away as quickly.

### EPAT-PERF.$^{CM}$.

Left ankle, hip, and shoulder pain him very much.

### FORMICA$^{45M}$.

Legs felt as if she had no power in them; sore; tired.

### GELSEM.$^{CM}$.

Thighs very sore (left mostly affected); very sensitive to touch; pains were all relieved when in perspiration.

### GETTYSBURG$^{45M}$.

Sharp pain in left knee.

### GLANDERINE$^{12}$.

Feet felt sore and tired, walking painful; soles of her feet very sensitive.

### HYPERICUM$^{45M}$.

Rapid cure of articular rheumatism; all the joints affected; much effusion about the knee-joints.

A female prover, after three days, found her feet *much swollen.*

Male prover. Sharp pains in the knees, could scarcely touch them, after taking the medicine for two weeks six times a day, this was the most prominent and severe symptom; his head symptoms were next in importance; dull pains shoot through his fingers as if they were festering.

In several provers appeared pains in the joints with some swelling like rheumatism of wrists, knees, and ankles.

### KALI-HYD.$^{CM}$.

Cured large, old, deep, foul looking syphilitic ulcers of the leg; verified a great many times.

Cured an old ulcer of the leg where the crude remedy had been given for a long time without any effect, and have had many cases the reverse of this, but do not know the philosophy or reason of it.

### LITHIA-CARB$^{5C}$.

Cured a deep, foul, old, ragged syphilitic ulcer on calf of leg in a short time.

Curative in old deep circular offensive varicose ulcers near the ankle, often associated with syphilis.

### MERC-VIV.$^{101M}$.

Much soreness in the thighs and arms, worse on pressure; motion is painful.

### MERC-IOD.$^{2C}$.

Thighs feel very much bruised, with general muscular soreness.

### NITRIC-ACID$^{5C}$.

Legs (mostly the left) very sore in front along the shin from ankle to knee; bound flannel on them to see if do any good. This symptom appeared in at least half-dozen provers, and has been often verified by cures.

### PETROL.$^{CM}$.

Knees feel very tired; walking is painful.

### PHYTOLACCA$^{47M}$.

Great pain down the sides of the hips and thighs.

### PLANTAGO-MIN.$^{11C}$.

*Suffering violently* with pain in her right thigh from her knee to the hip-joint and then around to the back, not constant pain, but it comes quick as thought, sometimes every few seconds and then perhaps twenty to thirty minutes apart, it is so severe that she *screams;* afterward there remains dull pain.

### POLYGONUM$^{42M}$.

From knees to her feet ached severely; aching pain in her legs prevented sleep; sore to touch.

Limbs ache and chilly sensations followed by fever mostly in the afternoon.

